# Multimodality Imaging Features of Papillary Renal Cell Carcinoma

**DOI:** 10.3390/diagnostics15070906

**Published:** 2025-04-01

**Authors:** Rosita Comune, Francesco Tiralongo, Eleonora Bicci, Pietro Paolo Saturnino, Francesco Michele Ronza, Chandra Bortolotto, Vincenza Granata, Salvatore Masala, Mariano Scaglione, Giacomo Sica, Fabio Tamburro, Stefania Tamburrini

**Affiliations:** 1 Department of Radiology, Ospedale del Mare-ASL NA1 Centro-Napoli, 80147 Naples, Italy; pietropsat@hotmail.it (P.P.S.); fabio.tamburro@aslnapoli1centro.it (F.T.); tamburrinistefania@gmail.com (S.T.); 2 Radiology Unit 1, Department of Medical Surgical Sciences and Advanced Technologies “GF Ingrassia”, University Hospital Policlinico “G. Rodolico-San Marco”, University of Catania, 95123 Catania, Italy; 3 Department of Radiology, Careggi University Hospital, 50134 Florence, Italy; 4 Department of Diagnostic Imaging, AORN “S. Anna e S. Sebastiano”, 81100 Caserta, Italy; francesco.ronza@virgilio.it; 5 Department of Clinical, Surgical, Diagnostic and Pediatric Sciences, University of Pavia, 27100 Pavia, Italy; chandra.bortolotto@unipv.it; 6 Department of Radiology, IRCCS Policlinico San Matteo, 27100 Pavia, Italy; 7 Radiology Division, Istituto Nazionale Tumori IRCCS Fondazione Pascale—IRCCS di Napoli, 80131 Naples, Italy; v.granata@istitutotumori.na.it; 8 Radiology Department of Surgery, Medicine and Pharmacy, University of Sassari, Viale S. Pietro, 07100 Sassari, Italy; samasala@uniss.it (S.M.); mscaglione@uniss.it (M.S.); 9 Department of Radiology, James Cook University Hospital, Marton Road Marton Rd., Middlesbrough TS4 3BW, UK; 10 Department of Radiology, Monaldi Hospital, 80131 Naples, Italy; gsica@sirm.org

**Keywords:** papillary renal cell carcinoma, ultrasound, tomography, magnetic resonance

## Abstract

**Objectives:** To describe the US, CEUS, CT, and MRI features of papillary renal cell carcinoma (PRCC) and to underline the imaging characteristics that are helpful in the differential diagnosis. **Methods:** Patients with histologically proven papillary renal cell carcinoma who underwent at least two imaging examinations (US, CEUS, CT, and MRI) were included in the study. Tumor size, homogeneity, morphology, perilesional stranding, contrast enhancement locoregional extension were assessed. A comparison and the characteristics of the imaging features for each imaging modality were analyzed. **Results:** A total of 27 patients with an histologically confirmed diagnosis of PRCC were included in the study. US was highly accurate in distinguishing solid masses from cystic masses, supporting the differential diagnosis of PRCC, as well as in patients with a poor representation of the solid component. CEUS significantly increased diagnostic accuracy in delineating the solid intralesional component. Furthermore, when using CEUS, in the arterial phase, PRCC exhibited hypo-enhancement, and in the late phase it showed an inhomogeneous and delayed wash-out compared with the surrounding renal parenchyma. At MRI, PRCC showed a marked restiction of DWI and was hypointense in the T2-weighted compared to the renal parenchyma. **Conclusions:** In our study, the characteristic hypodensity and hypoenhancement of PRCC make CT the weakest method of their recognition, while US/CEUS and MRI are necessary to reach a definitive diagnosis. Knowledge of the appearance of PRCC can support an early diagnosis and prompt management, and radiologists should be aware that PRCC, when detected using CT, may resemble spurious non-septate renal cyst.

## 1. Introduction

Renal cell carcinoma (RCC) is one of the top ten cancers affecting both men and women in the United States. RCC is a complex disease, with diverse inherited traits, molecular subtypes, and histology, and varies in clinical behavior and outcome [1,2,3]. Approximately 75% of renal cell carcinoma (RCC) cases are clear cell RCC (ccRCC). Approximately 10–20% of RCC cases are papillary RCC (pRCC) and the male-to-female ratio is greater than 1.5, with approximately 0.934 cases per 100,000 patient–years [3]. PRCC is more common in developed nations than in developing countries and its incidence, which is still increasing, varies globally (with a higher incidence in men of 77%) [4]. Metastatic pRCC (mPRCC) has a poor prognosis, potentially worse than that specified for metastatic ccRCC (mccRCC) [3].

The 2022 revision of the World Health Organization (WHO) Classification of Tumors of the Male Genital and Urinary Tract (5th edition) represents a substantial advance in understanding the morphologic, immunohistochemical, and molecular characteristics of renal tumors. This update integrates histopathologic assessment with immunohistochemistry (IHC) and molecular analyses, reflecting the growing influence of next-generation sequencing (NGS) in clinical practice. This molecularly informed approach improves diagnostic accuracy, allowing for the more effective classification and management of renal cell carcinoma (RCC) and other renal tumors [5,6,7].

A major change in the 2022 classification is the elimination of the subdivision of papillary RCC (pRCC) into types I and II, which had limitations in capturing the heterogeneity of these tumors. Instead, the updated system introduces additional morphological patterns, such as Warthin-type pRCC and low-grade oncocytic pRCC, along with genetic markers such as trisomies of chromosomes 7 and 17 and mutations in the MET gene. These refinements improve diagnostic accuracy and open avenues for targeted therapies [8,9].

The 2022 classification categorizes renal tumors into the following groups:

- Clear cell renal cell tumors.
- Papillary renal cell tumors.
- Oncocytic and chromophobe renal cell tumors.
- Collecting duct tumors.
- Other renal tumors.
- Molecularly defined cell renal cell carcinomas.

Among these, the group of “molecularly defined cell renal cell carcinomas” has been significantly expanded, reflecting advances in molecular research. A definitive diagnosis often requires the integration of phenotypic evaluation with IHC and confirmation of underlying genetic abnormalities [5,6,7,8,9,10].

By adopting a molecular approach, the 2022 WHO classification provides a more nuanced understanding of RCC heterogeneity, ensuring that treatment strategies are better tailored to the individual tumor subtype. This integration of molecular features into the diagnostic process marks a new era in the precise diagnosis and management of renal tumors.

In parallel with these advances, the widespread adoption of local and disease-sparing renal surgical techniques underscores the importance of accurate preoperative staging in RCC.

The increasing use of abdominal imaging modalities such as CT, MRI, and ultrasound has led to an increase in the incidental detection of small solid renal masses, including both benign and malignant lesions. This trend reflects not only technological advances, but also the increased public awareness of the importance of routine health screenings, allowing for early diagnosis and timely intervention. Consequently, RCC mortality rates have declined significantly in high-income countries over the past three decades, even as its overall incidence continues to increase [4,5,6,7]. However, the imaging evaluation of papillary renal cell carcinoma (pRCC) presents significant challenges due to its variable enhancement patterns, its overlapping features with other renal masses, and the limited specificity of conventional imaging techniques. Despite advancements in ultrasound, contrast-enhanced ultrasound (CEUS), computed tomography (CT), and magnetic resonance imaging (MRI), differentiating pRCC from other renal tumors remains difficult, often requiring histopathological confirmation after surgical resection.

In this article, we report the results of a four-year (1 January 2020–31 December 2024) retrospective study that aims to describe the imaging features of pRCC using ultrasound (US), contrast-enhanced ultrasound (CEUS), computed tomography (CT), and magnetic resonance imaging (MRI). We also want to highlight the imaging features that are crucial for the differential diagnosis of pRCC, focusing on how different imaging modalities contribute to identifying and differentiating pRCC from other renal lesions.

## 2. Materials and Methods

A four-year observational retrospective study was conducted at our institution (Department of Radiology, Ospedale del Mare-ASL NA1 Centro).

The inclusion criteria of our study were patients with histologically proven pRCC who underwent total or partial nephrectomy and who performed at least two imaging examinations (contrast enhanced CT, contrast-enhanced MRI, ultrasound, contrast-enhanced ultrasound).

We retrospectively reviewed records of all nephrectomies of renal masses performed at our institution from 2020 to 2024. All nephrectomies performed for benign masses and/or other malignancies were excluded from the study.

Demographic, clinical data, and imaging features such as tumor size, homogeneity, morphology, contrast enhancement, vein renal infiltration, lymph nodes involvement, metastasis, and number of renal arteries were evaluated.

The images were assessed by two radiologists with experience in urogenital imaging (15 and 10 years).

### 2.1. US and CEUS Technique

All conventional US and CEUS examinations were performed on high-end ultrasound systems equipped with up-to-date CEUS-specific protocols Samsung RS80A (Samsung Medison, Seoul, Corea del Sud).

Initial grayscale imaging was used to assess lesion location, size, and echogenicity, while color Doppler flow imaging (CDFI) evaluated blood flow in the lesions.

CEUS was performed after conventional US examination using the same scanning system: a second-generation blood-pool contrast agent (SonoVue^®^, Bracco, Milan, Italy) was administered via a peripheral 18–22 G needle, with a bolus injection of 1.6 to 2.4 mL of contrast agent followed by a 10 mL flush of 0.9% sodium chloride solution. During contrast imaging, a low mechanical index (<0.4) was consistently used to prevent premature microbubble destruction. CEUS videoclips were acquired during the examination to assess the arterial phase (10–30 s post-contrast injection) and the late phase (30–90 s post-contrast). To optimize image clarity, patients were asked to suspend breathing for up to 30 s during imaging; those with respiratory impairments were instructed to breathe lightly and slowly. The mean examination time ranged from 3 to 5 min.

All CEUS images and cine loops were digitally archived for further analysis. CEUS examinations were reviewed to ensure consistency and reliability.

The imaging features and characteristics analyzed at US and CEUS are reported in Table 1 and Table 2.

#### US and CEUS Image Analysis

The enhancement patterns observed on CEUS were categorized as follows:

- Wash-In Patterns: Contrast arrival in the lesion was classified as “synchronous-in” when the wash-in time matched that of the adjacent renal cortex, and “slow-in” when it was delayed.
- Washout Patterns: Contrast washout was categorized as “fast-out”, “synchronous-out”, or “slow-out” to indicate whether the contrast exited the tumor more quickly, simultaneously, or more slowly than from the surrounding cortex.
- Hypoenhancement refers to reduced contrast uptake compared to the surrounding renal parenchyma, isoenhancement indicates enhancement similar to the parenchyma, and hyperenhancement denotes greater contrast uptake, potentially reflecting differences in vascularity and tissue composition.
- Homogeneity at Peak Enhancement: Lesions showing uniform enhancement throughout, without any areas lacking contrast, were classified as having homogeneous enhancement. Conversely, a lesion was considered heterogeneous if it showed areas of varying contrast uptake, such as regions of hypoenhancement or non-enhancement mixed with iso- or hyperenhancing areas
- A rim of perilesional enhancement after SonoVue injection was considered to represent a pseudocapsule.

### 2.2. CT Technique

All CT examinations were performed with multiphase volume acquisition for renal disease (SOMATOM Drive dual-source computed tomography scanner, Siemens Healthineers, Erlangen, Germany). CT images were obtained while breath was being held with the following parameters: 120 kVp; 150–300 mA (depending on patient size); pitch, 0.75–1.5; section thickness, 1.25 mm; reconstruction interval, 2.5 mm. A non-contrast scan was performed to identify stones and intralesional fat and to determine the homogeneity and HU values of focal lesions. Next, intravenous contrast medium (1.0–1.5 mL/kg) was injected at 3–5 mL/s (possibly higher flows, depending on venous access), followed by 60 mL of an intravenous injection saline bolus. The corticomedullary phase was obtained with a delay of 25 s (threshold of 120 HU in the abdominal aorta before the origin of the renal artery). The nephrogenic phase was obtained with a delay of 90 s, to ensure optimal enhancement of the renal parenchyma and to emphasize the enhancement and washout of renal lesions. A delayed excretory phase was achieved after at least 8 min (400–500 s).

CTs were reviewed using digital archiving and reporting software (Carestream PACS 11.0) on axial and multiplanar reforms (MPR).

The imaging features and characteristics analyzed using CT are reported in Table 3.

#### CT Imaging Analysis

Regions of interest (ROIs) were identified and analyzed on both CT and MRI to evaluate tumor characteristics. For homogeneous lesions, the ROI was centrally placed within the tumor, ensuring consistent measurement. In cystic or necrotic lesions, the ROI was positioned in the solid tissue component, provided its size was sufficient for reliable evaluation. ROI placement was standardized in size and position across all imaging phases to ensure reproducibility.

To quantify tumor enhancement, the reviewer measured the attenuation values within the ROIs on CT images. The averaged measurements were used to improve accuracy. The ROI was carefully selected to cover the largest possible area of solid, enhancing tissue while excluding calcified regions.

Maximum enhancement, calculated as the difference between maximum density and pre-contrast density, represents the peak increase in density observed post-contrast.

Enhancement was deemed significant if the attenuation increased by 15–20 HU compared to non-enhanced images, whereas increases of less than 15 HU were interpreted as no enhancement.

### 2.3. MRI Technique

MRI was performed using a 1.5 T scanner (Amira, Siemens Medical Solutions, Erlangen, Germany) with a receive-only multichannel surface coil. MRI protocol included T1-weighted spin-echo (SE) sequences in the axial plane, half-Fourier acquisition single-shot turbo spin echo (HASTE) sequences in both the axial and coronal planes, and multi-shot turbo spin echo (BLADE) sequences in the axial plane. Diffusion-weighted images (DWI) with apparent diffusion coefficient (ADC) mapping were also acquired. Additionally, the standard MRI protocol for evaluating renal mass incorporated T2-weighted fast spin echo (FSE) sequences in multiple planes, as well as dynamic contrast-enhanced imaging in the arterial phase (30 s after beginning the injection), tubular phase (90 s), and delayed phase (3 to 6 min) to evaluate the vascularity and enhancing patterns of kidney injury.

The imaging features and characteristics analyzed using MRI are reported in Table 4.

#### MRI Analysis

Regions of interest (ROIs) were identified and analyzed using both CT and MRI to evaluate tumor characteristics. Using MRI, lesion signal intensity was assessed pre-contrast and at peak post-contrast phases. Percent enhancement was calculated using the formula: (maximum signal − signal without enhancement)/signal without enhancement × 100. An enhancement exceeding 15% was considered positive, a finding that correlates with the increased vascularity that is characteristic of certain neoplasms. This methodology was particularly useful in differentiating tumors with high vascularity from those with minimal enhancement, such as papillary renal cell carcinoma.

### 2.4. Statistic Analisys

Stata computer software version 17.0 (Stata Corp., College Station, TX, USA) was used for statistical analysis. Pearson’s χ^2^ test was used to evaluate pRCC lesions in terms of enhancement pattern, the homogeneity of enhancement, and the CT attenuation value and MVD. Continuous variables were expressed as mean values ± standard deviation, and discrete variables were expressed as numbers and percentages. Statistical significance was considered if *p* < 0.05.

## 3. Results

A total of 260 nephrectomies were performed during the study period; 80 histologically benign nephrectomies, either partially (18) or totally (61), were excluded from the study.

**Figure 1:**
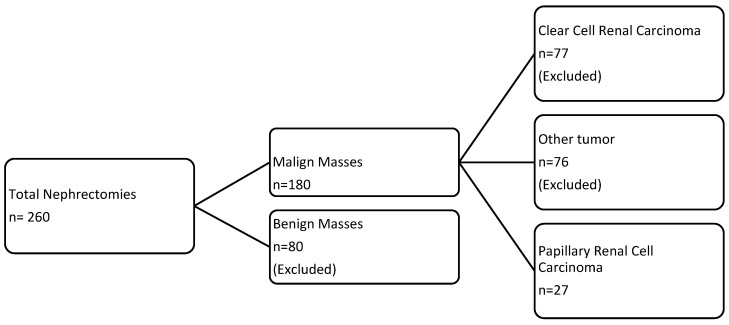
Flow diagram for patient selection in this retrospective study of papillary renal cell carcinoma.

Among the histologically malignant nephrectomy, 77 were clear cell renal tumors, 76 were excluded because their histology showed different isotypes (including chromophobe, urothelial, transitional cell urothelial, lymphoma, and liposarcoma). Twenty-seven patients were histologically confirmed to have papillary renal cell carcinoma (Figure 1).

Our study cohort was therefore composed of 27 patients (7 partial nephrectomies), with an average age at the time of surgery of 63.2 years. The cohort demonstrated a male predominance, with 83.3% of cases occurring in males and 16.7% in females.

In our cohort, most tumors were encapsulated, with 92.6% exhibiting a well-defined capsule. Only two tumors (7.4%) displayed an infiltrative growth pattern. Additionally, one case presented with necrotic enlarged retroperitoneal lymph nodes (in the peri-hilar and inter-aortocaval regions).

The lesions were homogeneous in 85.2% of cases, with an average size of 29.4 mm for the cystic or necrotic components.

Calcifications were present in 7.4% of tumors (*n* = 2), with half of these displaying peripheral, linear calcifications and the other half exhibiting a rounded appearance (Table 5).

### 3.1. Ultrasound (US)

**Figure 2:**
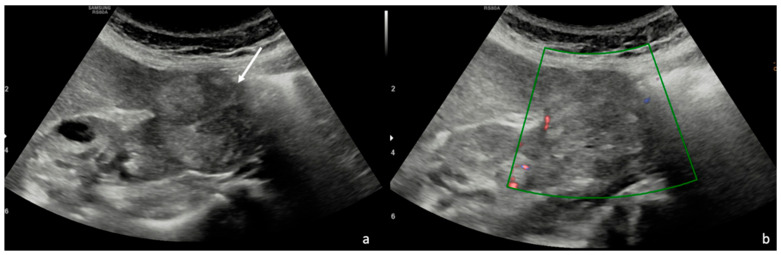
(**a**) B-mode ultrasound shows an exophytic solid renal lesion (white arrow). (**b**) Color–Doppler shows no intralesional significant vascularization.

In our study, of the majority of pRCCC patients (70.3%, *n* = 19/27) showed hypoechoic features; inhomogeneous hypoechoic lesions were less common and were identified in only 11.1% of patients (*n* = 3). In 18.5% of cases (*n* = 5) pRCC was observed. Intralesional calcifications were observed in 7.4% of patients (*n* = 2), but were not perfectly clear. Tumor vascularization was noted in 37% (*n* = 10) of cases. The US findings are summarized in Table 6 (Figure 2).

### 3.2. Contrast-Enhanced Ultrasound (CEUS)

**Figure 3:**
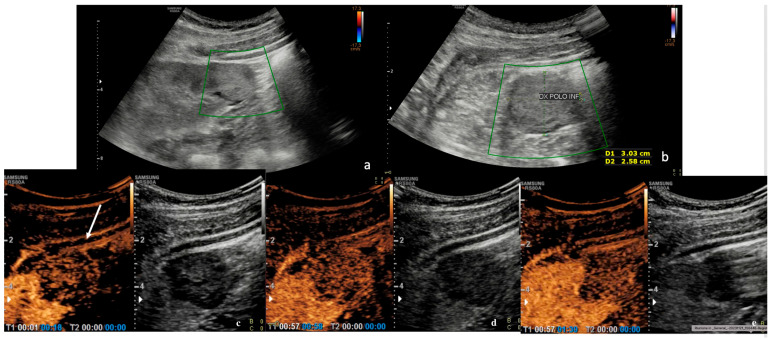
(**a**,**b**) B-mode ultrasound shows a solid exophytic hyperechoic renal lesion at the right lower renal pole. CEUS. During the arterial phase (**c**) (18 s), the lesion appeared hypovascular compared to adjacent renal parenchyma. (**d**,**e**) In the venous and late phase (58 s to 1.30 min), the lesion showed rapid and inhomogeneous wash-out compared to the adjacent renal parenchyma (white arrow).

At CEUS, 70.3% of patients (*n* = 19) showed a slow enhancement pattern. Instead, a simultaneous wash-in pattern, involving the concomitant absorption of contrast by the lesion and the surrounding renal parenchyma, was observed in 29.7% of cases (*n* = 8). A fast wash-in pattern was not assessed. In terms of contrast wash-out pattern, most tumors (81.5%, *n* = 22) showed a fast-wash out pattern. A simultaneous washout was observed in 11.1% of cases (*n* = 3), while a slow-out pattern was present in 7.4% of cases (*n* = 2). When examining the levels of contrast enhancement, 52.2% of tumors showed hypoenhancement (*n* = 12), while 29.6% showed isoenhancement (*n* = 8) and 25.9% showed hyperenhancement (*n* = 7). In CEUS, a lesion is defined as homogeneous when it exhibits a uniform contrast enhancement throughout its structure, without visible differences in echogenicity. At CEUS, 62.9% of lesions appeared to be homogeneous (*n* = 17) and 37% appeared to be heterogeneous (*n* = 10). The CEUS findings are presented in Table 6 (Figure 3).

### 3.3. CT

#### Density and Contrast Enhancement

In our sample of 27 patients, the mean pre-contrast tumor density was 30.3 HU (20–38 HU).

Density changes were calculated for all lesions after intravenous contrast administration in all phases (basal, arterial, venous, and late). Subjectively, no appreciable enhancement of the lesion was observed in 12 out of 27 patients (44.4%). Among these, the ROI measures of density change were between 10 and 19 HU 18.5% (*n* = 5); these five lesions did not show significant post-contrast enhancement.

**Figure 4:**
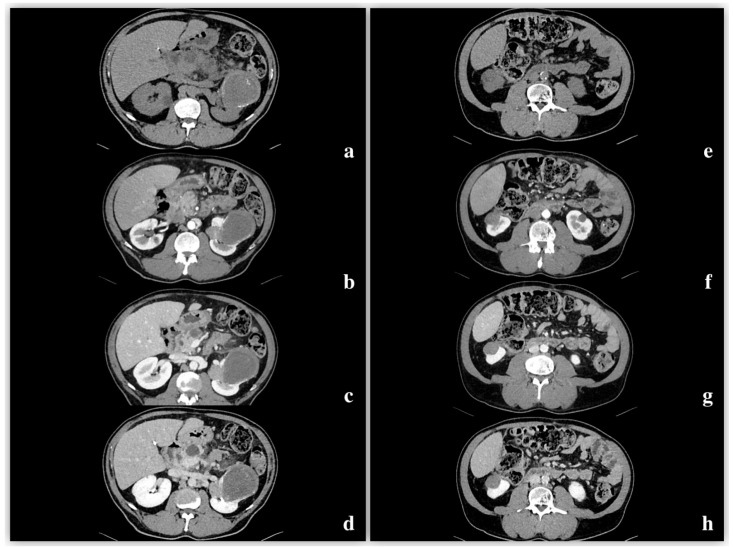
Patient 1 (**right** image) (**e**–**h**): On unenhanced CT, a slightly hypodense lesion (35 HU) with calcifications was detected along the anterior margin of the left kidney. During the corticomedullary phase (CMP), the lesion exhibited minimal enhancement (45 HU). In the venous phase, the lesion was increased to 68 HU, showing no significant washout but presenting hypodense areas within. In the excretory phase, there was no substantial washout except for a centrally located hypodense area (60 HU). Patient 2 (**left** image) (**a**–**d**): A papillary renal lesion located at the down pole of the right kidney is observed. Across all phases of the CT study, the lesion exhibits attenuation characteristics similar to those of a renal cyst, demonstrating a homogeneous, hypodense appearance with no significant enhancement or washout.

The mean tumor densities in the different CT phases were as follows (Figure 4):

- Pre-enhanced phase: 30.3 ± 6.5 HU.
- Arterial phase: 47.2 ± 12.4 HU.
- Venous phase: 64.6 ± 17.3 HU.
- Delayed phase: 61.8 ± 14.5 HU.

**Figure 5:**
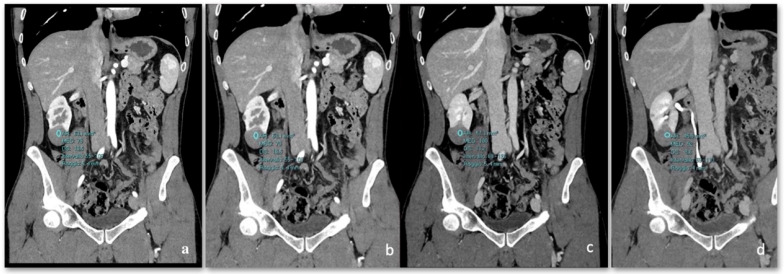
(**a**) Coronal non-enhanced CT shows a slightly hyperdense round lesion at the lower pole of the right kidney (36 HU). (**b**) In the arterial phase, the lesion shows a minimal enhancement of 79 HU. (**c**) In the nephrogenic phase, minimal and delayed enhancement is observed, reaching 100 HU. (**d**) In the delayed phase, the lesion shows no significant washout at 92 HU.

One tumor contained adipose tissue (density less than −20 HU) histologically due to bone metaplasia and calcifications. In one case, we obtained different data showing a pre-enhanced density of 36 HU; in the arterial phase, an enhancement of 79 HU was reached, followed by 100 HU in the nephrogenic phase, with no significant washout (92 HU) in the delayed phase (Figure 5).

### 3.4. MRI

#### Signal Intensity, Homogeneity, and Contrast Enhancement

**Figure 6:**
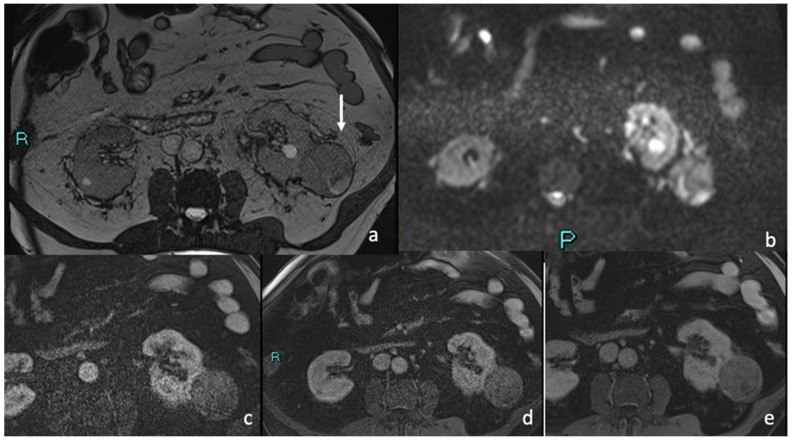
Axial T2-weighted (**a**) right cortical mesorenal mass with well-defined margins hypointense on T2-weighted image ((**a**), as shown by the arrow). (**b**) When using diffusion-weighted spin-echo imaging (DWI), the lesion show restricted diffusion, allowing for a correct differential diagnosis from a hemorrhagic cyst. In the postcontrast image (arterial phase (**c**), venous phase (**d**), and delay phase (**e**)), there is minimal, faint, irregular, spot-like enhancing foci within the lesion, with no considerable washout.

At MRI, the majority of tumors (85.2%) showed a homogeneous signal. A lower percentage was slightly heterogeneous (14.8%). Compared to normal renal parenchyma, the majority of tumors (88.9%) were hypointense on T2-weighted images, while 7.4% appeared hyperintense and 3.7% were isointense. T1-weighted images revealed a greater variability in signal intensity: 59.5% of tumors were isointense, 29.6% were hypointense, and 11.1% were hyperintense compared to surrounding renal tissue. All tumors showed marked hyperintensity on DWI sequences and hypointensity on ADC maps due to marked restriction (Table 7) (Figure 6).

All analyzed tumors showed substantial enhancement upon MRI, and contrast enhancement was observed in 80% of cases. Enhancement exceeding the 15% threshold was classified as positive. This threshold allowed for a more precise assessment of the vascular characteristics of the tumors, providing valuable information on their behavior and aiding in the differentiation of neoplastic subtypes. The heterogeneity of enhancement observed among the lesions strongly reflected their underlying histological characteristics, underscoring the value of MRI in capturing these critical tumor-specific features.

### 3.5. Technique Comparison

The results highlight the main characteristics of PRCCs and the strengths of each technique in identifying key tumor-associated features. The main feature being analyzed, such as encapsulated growth pattern, heterogeneity/homogeneity, tumor size, contrast enhancement, tissue characteristics (differentiation between solid, cystic, necrotic, and vascular components), renal vein invasion and necrotic lymph nodes, were compared for the different imaging techniques.

US proved to be useful in identifying the basic echogenic characteristics of renal tumors, such as hypoechoic, inhomogeneous hypoechoic, and hyperechoic lesions. Although less detailed than CT or MRI, ultrasound provided valuable information on lesion contours and their relationship with the surrounding renal parenchyma, making it a useful initial screening tool.

CEUS was highly useful in detecting contrast enhancement dynamics, particularly in differentiating hypo-, iso-, and hyper-enhancement patterns. Due to its real-time visualization of blood flow and vascular enhancement, CEUS provided an additional diagnostic perspective for evaluating the vascular characteristics of renal papillary tumors, especially for smaller lesions or those with low vascularity.

CT was excellent for assessing morphological features such as tumor encapsulation, growth pattern, calcifications, tumor size, and the presence of fatty tissue. It was also effective in detecting vascular invasion and necrotic lymph nodes, which are critical for tumor staging. CT’s ability to visualize these morphological characteristics allowed for a detailed assessment of tumor boundaries and its impact on the surrounding renal parenchyma. In CT imaging, papillary renal carcinoma often presents as an indeterminate lesion or can mimic pseudocysts, posing a significant diagnostic challenge. This limitation of CT underscores the difficulty in distinguishing papillary renal carcinoma from benign lesions, highlighting the need for complementary imaging modalities to improve diagnostic accuracy.

**Figure 7:**
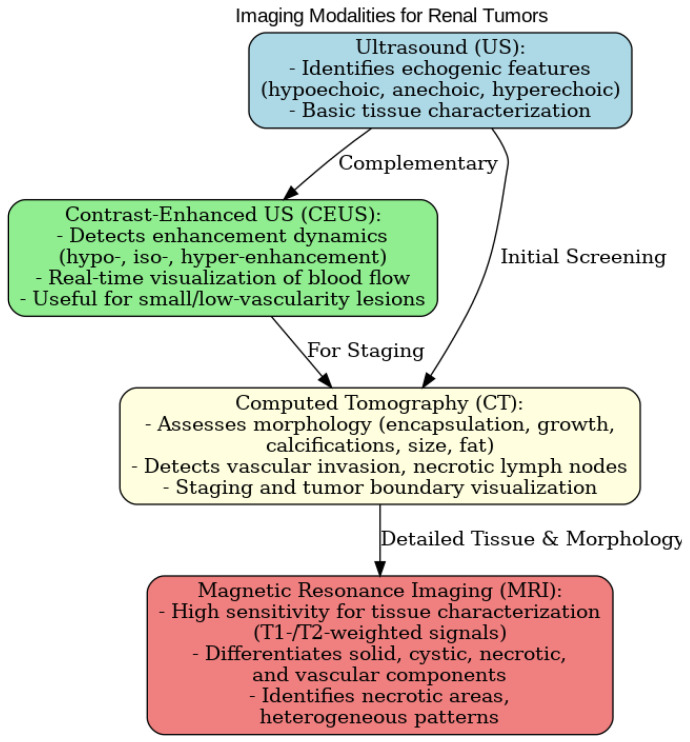
Summary of pRCC main features and of the different imaging modalities for the diagnosis of pRCC, shown via flow-chart.

**Figure 8:**
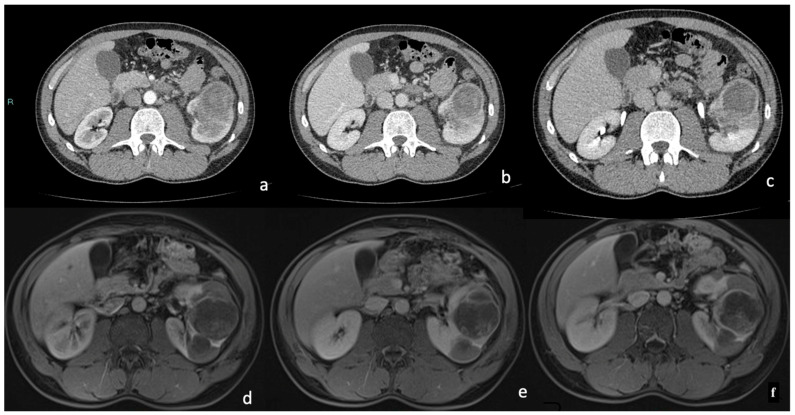
Enhanced CT in the arteriosus phase: (**a**) A hypodense renal lesion was detected on the anterior margin of the left kidney exophytic (<25%) with thick enhancing walls. (**b**) In the venous phase, and delayed phase (**b**,**c**) slightly intralesional enhancing was appreciable, with no clear detection of intralesional solid component. The lesion did not show significant washout, either centrally or peripherally. Enhanced MRI image in the arteriosus phase: (**d**) The left cortical renal mass appeared well-defined and hypointense. In the post-contrast image ((**e**), venous phase), irregular intralesional enhancing foci were present; in the delay phase (**f**), the lesion did not show considerable washout.

**Figure 9:**
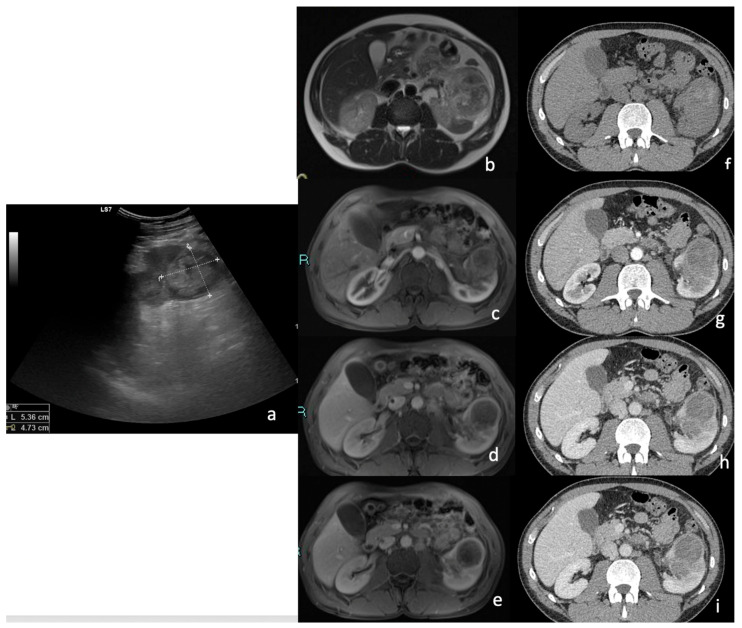
(**a**) B-mode ultrasound showed an inhomogeneous hyperechoic exophytic renal lesion. The axial T2-weighted image (**b**) shows a left cortical, exophytic, interpolar renal mass that was inhomogeneous and moderately hypointense in the T2-weighted image; in the axial post-contrast images, in the (**c**) arteriosus phase (**d**), venous phase, and (**e**) delay phase, the lesion was homogenously enhanced and there was no significant washout in the delayed phase. Axial unenhanced (**f**) and arteriosus-, venous-, and delay-phase (**g**–**i**) CT images showed a complex mass in left kidney with intralesional high-density foci in non-contrast images. After contrast, the lesion did not show significant enhancement.

MRI has proven to be particularly useful in evaluating tissue characteristics, particularly in terms of signal intensity on T1- and T2-weighted images, as well as in assessing dynamic enhancement patterns following contrast administration. These features make MRI particularly suited for detailed tissue characterization, enabling differentiation between solid, cystic, necrotic, and vascular components within the tumor. MRI was especially effective in identifying necrotic areas and heterogeneous enhancement patterns, as are commonly observed in renal papillary tumors (Figure 7, Figure 8 and Figure 9).

## 4. Discussion

Renal cell carcinoma (RCC) has become more common worldwide over the past 20 years, primarily due to the proliferation of more non-invasive abdominal imaging methods such as MRI, CT, CEUS, and US. Most RCCs are now discovered incidentally as small tumors in patients who have no symptoms; this has significant implications for the management of renal tumors as it requires a cautious approach to diagnosis and treatment [6,7]. The rate of incidental detection has increased from 7–13% in the 1970s to 48–66% today [1,5].

Because patients are often surgically treated with partial or radical nephrectomy soon after diagnosis, the natural history of small renal tumors is not well studied. However, previous research has shown that localized tumors grow more slowly than advanced or metastatic tumors, and retrospective investigations have shown that tumors ≤ 3 cm rarely spread and grow slowly. Furthermore, while most small tumors grow slowly or not at all, those that are predicted to grow rapidly and spread typically do so early.

A variety of histological, immunohistochemical, and cytogenetic features form the basis of the new histopathological classification of RCC. These features allow one to distinguish between the various subtypes of RCC, which mainly consist of chromophobe renal cell carcinoma (chRCC), pRCC, and ccRCC. Understanding these subtypes is necessary to determine the best management plans for patients with renal tumors. Approximately 10–15% of all RCC cases are pRCC, making this a relatively frequent RCC subtype [1,2]. Its prevalence highlights the importance of early and correct diagnosis, which is essential for successful treatment. To differentiate pRCC from other benign and malignant renal diseases, early diagnosis is crucial. It is essential to distinguish between benign and malignant lesions since nephron-sparing surgery or radical nephrectomy are not recommended for the treatment of benign tumors, and management and patient outcomes are significantly affected by this distinction [7,8,9,10].

Compared to clear cell renal cell carcinoma (cRCC), papillary renal cell carcinoma (pRCC) has reduced vascularity, which accounts for its decreased computed tomography (CT) enhancement. Microvessel density (MVD) differences account for this discrepancy; pRCC has substantially less tiny vasculature than cRCC. 

pRCC usually exhibits less enhancement than cRCC on contrast-enhanced CT in all imaging phases, but especially in the corticomedullary phase. Despite having an identical vascularity, pRCC and chromophobe RCC can be distinguished from one another through differences in enhancement. Although they are a little more frequent in pRCC than in cRCC, calcifications are not a trustworthy indicator of difference.

Although solid pRCCs are often homogeneous, particularly when they are less than 3 cm in size; bigger tumors may show heterogeneity and necrosis. Cystic pRCCs, which have characteristics like a unilocular cyst with a tumor nodule, can develop from the inherent architecture or because of necrosis. 

When distinguishing between pRCC and cRCC, magnetic resonance imaging (MRI) may be helpful, especially for individuals who are unable to tolerate iodinated contrast. Compared to cRCC, pRCC displays less strong enhancement and a lower signal intensity on both T1- and T2-weighted imaging. The subtle differences in enhancement may be further clarified via MRI subtraction techniques. 

Although less frequently utilized, ultrasound (US) is nevertheless useful for differentiating between solid and cystic masses, particularly when there borderline enhancement is observed following CT. Like angiomyolipomas, small pRCCs might appear hyperechogenic, emphasizing the need for additional testing [11,12,13,14,15,16].

The primary objective of this study was to comprehensively describe the imaging features associated with papillary renal cell carcinoma using various modalities, including US, CEUS, CT, and MRI, and to understand the key features in the identification of the latter and the sensitivity and diagnostic value of the modalities used to identify the key features. It also sought to determine how the combined use of these imaging techniques could improve diagnostic accuracy in identifying pRCC.

Our results highlighted the main characteristics of PRCCs and the strengths of each technique in identifying key tumor-associated features. The main feature analyzed, such as encapsulated growth pattern, heterogeneity/homogeneity, tumor size, contrast enhancement, tissue characteristics (differentiation between solid, cystic, necrotic, and vascular components), renal vein invasion, and necrotic lymph nodes, were compared when using the different imaging techniques. In this study, both US and CEUS emerged as the most useful tools for characterizing renal masses. Ultrasound played a crucial role in distinguishing between solid and cystic lesions, especially in cases where the CT findings were inconclusive. US proved to be useful in identifying the basic echogenic characteristics of renal tumors, such as hypoechoic, anechoic, and hyperechoic lesions. Although less detailed than CT or MRI, ultrasound provided valuable information on lesion contours and the lesion’s relationship with the surrounding renal parenchyma, making it a useful initial screening tool.

CEUS further enhanced this diagnostic ability, clarifying the nature of complex cystic masses by effectively distinguishing solid components from debris or blood clots, distinctions that can be difficult to achieve with conventional CT or ultrasound techniques. Furthermore, the ability of CEUS to visualize tumor microvasculature demonstrated greater sensitivity than CT, revealing the blood flow in all pRCC lesions even when CT showed suspicious or no enhancement. During the arterial phase of CEUS, papillary renal cell carcinoma (pRCC) typically showed hypo-enhancement, reflecting its reduced vascularity compared to the surrounding renal parenchyma. This pattern of hypo-enhancement, combined with rapid contrast washout during the late phase, significantly supported the diagnosis of malignancy [17,18,19,20,21]. In our study, CEUS was shown to be highly useful in detecting contrast enhancement dynamics, particularly in differentiating hypo-, iso-, and hyper-enhancement patterns. Due to its real-time visualization of blood flow and vascular enhancement, CEUS provided an additional diagnostic perspective for evaluating the vascular characteristics of renal papillary tumors, especially smaller lesions or those with low vascularity. An additional advantage of CEUS is its safety profile; it can be used in patients with renal insufficiency due to its non-nephrotoxic contrast agents and the absence of ionizing radiation, making it a viable option for a broader patient population. Our findings are consistent with those of Xue et al., who demonstrated that CEUS features such as slow wash-in, rapid wash-out, and hypoenhancement at peak are characteristic of pRCC [22,23,24].

When assessed via computed tomography (CT), pRCC typically appeared as hypodense lesions on nonenhanced scans, showing minimal enhancement (10–30 Hounsfield units) after the administration of contrast agents. A significant diagnostic challenge arises due to the similarity of pRCC to hyperdense cysts, which complicates the differentiation between benign and malignant lesions. This challenge is particularly pronounced in small tumors (<3 cm) or those with poor intertumoral vascularization, where CT often misrepresented pRCC as hyperdense cysts, leading to potential misdiagnosis. In this context, MRI has emerged as the most effective modality to characterize pRCC. The use of diffusion-weighted imaging (DWI) sequences has shown high sensitivity in identifying pRCC, which has shown marked restrictions indicative of high cellular density. Furthermore, T2-weighted images have provided a further means of differentiating pRCC, showing its lesions to be hypointense compared to renal parenchyma and accurately identifying solid intralesional components, a critical aspect of differential diagnosis. The presence of cystic or necrotic features in tumors increases the complexity of the diagnostic process, particularly when differentiating pRCC from clear cell carcinoma. In addition, atypical tumors that may contain fat or exhibit infiltrative features present additional diagnostic challenges that must be carefully addressed. The absence of fat-containing features on MRI also helped to differentiate pRCC from other renal tumors, such as angiomyolipomas. MRI ultimately excelled in detecting subtle intra-tumoral enhancement, particularly when using subtraction imaging techniques, and demonstrated superior sensitivity in detecting contrast enhancement compared to CT in all lesions that did not show significant enhancement on CT. The study ultimately concluded that pRCC typically presents as a small, homogeneous, hypovascular, and noncalcified tumor that appears hypointense on T2-weighted sequences. It is noteworthy that the density change was indeterminate in 10% of cases and negative in 5% of tumors on CT, underlining the importance of careful interpretation to avoid misclassifying these tumors as cystic lesions. In case of diagnostic uncertainty, MRI should be performed because of its superior sensitivity to contrast enhancement and its superior ability to recognize the compositional features of the lesion. Our results are in line with those of Couvidat et al., who demonstrated that pRCC typically appears as a homogeneous, encapsulated tumor with low enhancement on CT and hypointense signals on T2-weighted MRI sequences. These shared imaging features underscore the complementary utility of CT and MRI in characterizing pRCC and distinguishing it from other renal tumor subtypes [25].

Several studies have shown that MRI using gadolinium-based contrast agents provides an improved contrast resolution, which improves tissue characterization and the detection of smaller or more heterogeneous lesions, particularly in tumors with little vascular involvement, features that may be missed by CT due to its lower contrast resolution [15,16]. Additionally, MRI demonstrated fewer beam-hardening artifacts, a common problem with CT, improving image quality, particularly in fatty regions or tumors close to bone. In conclusion, while CT remains a valuable diagnostic tool, MRI offers significant advantages in the evaluation of papillary renal tumors due to its superior tissue characterization, better assessment of vascular involvement, and ability to detect smaller or more heterogeneous lesions [26,27].

The ROI placement method is a limitation in this study, as well as in other studies attempting to quantify enhancement with CT and MRI. Although our study employed single-slice ROI measurements, a technique commonly used in both clinical practice and previous research [28,29,30], it could be argued that continuous sampling across the tumor parenchyma in multiple axial slices [30,31,32,33,34,35,36,37,38,39,40,41,42,43,44,45,46] may have further minimized the number of indeterminate enhancing pRCCs.

## 5. Conclusions

Our study showed the main features of each imaging technique in the diagnosis of pRCC, highlighting the most useful characteristics of each imaging modality that may improve the diagnostic accuracy. PRCC at CT can appear as a substantially homogeneous hypodense lesion and the solid intralesional component can be difficult to identify. Other imaging modalities should be performed to correctly identify PRCC, especially because it can appear at CT as a hyperdense cyst. US and CEUS are widely available and can identify the solid intralesional component with high accuracy. MRI was the most effective diagnostic modality in characterizing renal masses; contrast-enhanced MRI may be more advantageous than CT as the subtraction pictures can occasionally reveal papillary enhancement. In conclusion, in our study, the characteristic hypodensity of PRCC meant that CT was the weakest method in terms of recognition, and the use of US/CEUS and MRI is necessary to reach a definitive diagnosis. Knowledge of the appearance of PRCC can support early diagnosis and prompt management.

In conclusion, each imaging technique exhibited complementary strengths in identifying and characterizing renal papillary tumors. CT proved particularly useful for morphological assessment, MRI excelled in tissue characterization and enhancement pattern evaluation, US was valuable for basic echogenicity and lesion contours, and CEUS provided detailed information on vascular dynamics. Combining these techniques can significantly enhance diagnostic accuracy in the management of renal papillary carcinoma. Overall, our findings suggest that CT alone is insufficient for a definitive diagnosis, and integrating US, CEUS, and MRI is essential for improving diagnostic accuracy. Recognizing the imaging characteristics of pRCC is crucial for early diagnosis and appropriate management, ultimately aiding in better clinical decision-making.

Although our findings provide valuable insights into the imaging characteristics of papillary renal cell carcinoma (pRCC), they should be interpreted with caution and require further confirmation through larger, multicenter studies to strengthen their clinical applicability.

## Figures and Tables

**Table 1:** US lesion characteristics.

Parameter	Description
Topography	- Number of tumors: Single or multiple. - Location: Tumor’s position in the kidney. - Size: Measured using 2D imaging in the axial plane.
Morphology/Tumor Borders	- Encapsulated: Well-defined, hypoechoic borders with mass effect on adjacent structures. - Infiltrative: Ill-defined margins without altering the kidney’s outline, may invade surrounding tissues
Echogenicity	Internal echogenicity classified as follows: - Hypoechoic. - Inhomogeneous hypoechoic. - Hyperechoic. - Isoechoic (relative to renal parenchyma).
Homogeneity	Uniformity of the tumor’s echo pattern:
	Homogeneous vs. Heterogeneous.
Locoregional Extension	- Renal vein invasion: Changes in caliber and echogenicity of the renal vein. - Lymph nodes: Enlarged, hypoechoic structures near the kidney.

**Table 3:** CT lesion characteristics.

Parameter	Description
Topography	- Number of tumors: Single or multiple. - Location: Position in the kidney. - Size: Measured in two perpendicular axes (axial plane).
Morphology/Tumor Borders	- Encapsulated: Well-defined boundaries, causing deformation of the kidney contour and exerting a mass effect on adjacent structures. - Infiltrative: Poorly defined boundaries, no change to kidney contour but potential enlargement through adjacent structure invasion.
Density Measurement	Measurement in Hounsfield units (HU) in pre-contrast, arterial, venous, and late phases. Additional Features: - Calcifications. - Fatty Components: Density < −20 HU. - Homogeneity after iodine contrast.
Homogeneity	Uniformity of the contrast distribution after washout: homogeneous vs. heterogeneous.
Locoregional Extension	- Renal Vein Invasion. - Lymph Nodes: Presence of enlarged lymph nodes.

**Table 4:** MRI lesion characteristics.

Parameter	Description
Topography	- Number, location, and size (measured in two perpendicular dimensions). - Tumor boundaries and relationship to surrounding structures based on signal intensity variations.
Morphology/Tumor Borders	- Encapsulated: Well-defined boundaries, causing deformation of the kidney contour and exerting a mass effect on adjacent structures. - Infiltrative: Poorly defined boundaries, no change to kidney contour but potential enlargement through adjacent structure invasion.
Signal Intensity and Homogeneity	Assessed on T1 and T2 sequences (hypotense, hyperintense or isointense compared to renal parenchyma). Homogeneity assessed paying attention to regions of necrosis, cystic degeneration, or hemorrhage (low signal on T2). Hyperintense on DWI sequences and hypointense on ADC maps, indicating marked restriction.
Contrast Enhancement	Enhancement measured across dynamic phases. Pre-contrast and peak post-contrast signal intensity.
Locoregional Extension	- Assessed for renal vein invasion and lymph node enlargement. - Abnormal signal intensities used to identify pathological enlargement.

**Table 2:** CEUS lesion characteristics.

Parameter	Description
Topography	- Number of tumors: Single or multiple. - Location: Tumor’s position in the kidney. - Size: Measured on two perpendicular axes (axial plane).
Morphology/Tumor Borders	- Encapsulated: Well-defined borders with mass effect on adjacent structures. - Infiltrative: Poorly defined margins, no change to kidney contour, may expand through adjacent structure invasion.
Enhancement	Dynamic Enhancement: Patterns observed during wash-in and wash-out phases. Degree of enhancement during contrast phases: - Hypo-enhancement. - Iso-enhancement. - Hyper-enhancement (at peak). Hypovascular Areas: - Suggestive of necrosis or cystic degeneration.
Homogeneity	Uniformity of the contrast distribution after washout: Homogeneous vs. Heterogeneous.
Locoregional Extension	- Renal vein invasion: Assessed via contrast-enhanced abnormal vascularity. - Lymph nodes: Enlarged nodes with abnormal vascularity.

**Table 5:** Characteristics.

Features	%	*n*.	*p* Value
Encapsulated growth pattern	92.6%	25	***p* < 0.05**
2. Infiltrative growth pattern	7.4%	2	*p* > 0.05
3. Homogeneous lesions	85.2%	23	***p* < 0.05**
4. Heterogeneous lesions	14.8%	4	*p* > 0.05
5. Calcifications	7.4%	2	*p* > 0.05

**Table 6:** Ultrasound (US) characteristics and contrast-enhanced ultrasound (CEUS) characteristics.

US Features	%	*n*.	*p* Value
Hypoechoic	70.3%	19	***p* < 0.05**
2. Inhomogeneous Hypoechoic	11.1%	3	
3. Hyperechoic	18.5%	5	*p* > 0.05
4. Vascularity	37.0%	10	*p* > 0.05
**CEUS Features**	**%**	***n*.**	*p* > 0.05
Wash-In Slow	70.3%	19	***p* < 0.05**
2. Wash-In Simultaneous	29.7%	8	*p* > 0.05
3. Wash-In Fast	0%	0	*p* > 0.05
4. Enhancement Level: Hypo	52.2%	12	***p* < 0.05**
5. Enhancement Level: Iso	29.6%	8	*p* > 0.05
6. Enhancement Level: Hyper	25.9%	7	*p* > 0.05
7. Homogeneous Enhancement	62.9%	17	*p* > 0.05
8. Heterogeneous Enhancement	37.0%	10	*p* > 0.05
9. Washout Fast	81.5%	22	***p* < 0.05**
10. Washout Simultaneous	11.1%	3	*p* > 0.05
11. Washout Slow	7.4%	2	*p* > 0.05

**Table 7:** MRI features.

MRI FEATURES	%	*n*.	*p* Value
1. T2-Weighted Signal: Homogeneous Heterogeneous	85.2% 14.8%	23 4	***p* < 0.05** *p* > 0.05
2. T2-Weighted Signal Hypointense Hyperintense Isointense	88.9% 7.4% 3.7%	24 2 1	***p* < 0.05** *p* > 0.05 *p* > 0.05
3. T1-Weighted Signal Hypointense Hyperintense Isointense	29.6% 11.1% 59.5%	8 3 16	*p* > 0.05 *p* > 0.05 ***p* < 0.05**
4. DWI Hyperintense and Hypointense in ADC Maps	100%	27	***p* < 0.05**

## Data Availability

The original contributions presented in this study are included in the article. Further inquiries can be directed to the corresponding authors.

## References

[1] Sweeney P.L., Jang A., Halat S.K., Pal S.K., Barata P.C. (2022). Advanced papillary renal cell carcinoma: Epidemiology, genomic drivers, current therapies, and ongoing trials. Cancer Treat. Res. Commun..

[2] Tamburrini S., Consoli L., Garrone M., Sfuncia G., Lugarà M., Coppola M.G., Piccirillo M., Toto R., Stella S.M., Sofia S. (2022). The “Black Pattern”, a Simplified Ultrasound Approach to Non-Traumatic Abdominal Emergencies. Tomography.

[3] Al-Marhoon M.S. (2010). Small Incidental Renal Masses in Adults: Review of the literature. Sultan Qaboos Univ. Med. J..

[4] Ullah A., Yasinzai A.Q.K., Daino N., Tareen B., Jogezai Z.H., Sadia H., Jamil N., Baloch G., Karim A., Badini K. (2023). Papillary Renal Cell Carcinoma: Demographics, Survival Analysis, Racial Disparities, and Genomic Landscape. J. Kidney Cancer VHL.

[5] Volpe A., Panzarella T., Rendon R.A., Haider M.A., Kondylis F.I., Jewett M.A. (2004). The natural history of incidentally detected small renal masses. Cancer.

[6] Mousavi S.E., Najafi M., Aslani A., Fazlollahi A., Yekta Z., Sadri M., Nejadghaderi S.A. (2024). A population-based study on incidence trends of kidney and renal pelvis cancers in the United States over 2000–2020. Sci. Rep..

[7] Bahadoram S., Davoodi M., Hassanzadeh S., Bahadoram M., Barahman M., Mafakher L. (2022). Renal cell carcinoma: An overview of the epidemiology, diagnosis, and treatment. G Ital. Nefrol..

[8] Chow W.H., Dong L.M., Devesa S.S. (2010). Epidemiology and risk factors for kidney cancer. Nat. Rev. Urol..

[9] Goswami P.R., Singh G., Patel T., Dave R. (2024). The WHO 2022 Classification of Renal Neoplasms (5th Edition): Salient Updates. Cureus.

[10] Alaghehbandan R., Siadat F., Trpkov K. (2022). What’s new in the WHO 2022 classification of kidney tumours?. Pathologica.

[11] Millet I., Doyon F.C., Hoa D., Thuret R., Merigeaud S., Serre I., Taourel P. (2011). Characterization of small solid renal lesions: Can benign and malignant tumors be differentiated with CT?. AJR Am. J. Roentgenol..

[12] Vikram R., Ng C.S., Tamboli P., Tannir N.M., Jonasch E., Matin S.F., Wood C.G., Sandler C.M. (2009). Papillary renal cell carcinoma: Radiologic-pathologic correlation and spectrum of disease. Radiographics.

[13] Comune R., Grassi F., Picchi S.G., De Simone F., Sarti G., Giardina C., Galluzzo M., Scaglione M., Tamburrini S. (2024). Gross hematuria: Renal cell carcinoma mimicking a renal arteriovenous malformation. Radiol. Case Rep..

[14] Hollingsworth J.M., Miller D.C., Daignault S., Hollenbeck B.K. (2006). Rising incidence of small renal masses: A need to reassess treatment effect. J. Natl. Cancer Inst..

[15] Remzi M., Özsoy M., Klingler H.C., Susani M., Waldert M., Seitz C., Schmidbauer J., Marberger M. (2006). Are small renal tumors harmless? Analysis of histopatho- logical features according to tumors 4 cm or less in diameter. J. Urol..

[16] Frank I., Blute M.L., Cheville J.C., Lohse C.M., Weaver A.L., Zincke H. (2003). Solid renal tumors: An analysis of pathological features related to tumor size. J. Urol..

[17] Li G., Cuilleron M., Gentil-Perret A., Tostain J. (2004). Characteristics of image-detected solid renal masses: Implication for optimal treatment. Int. J. Urol..

[18] Chow W.H., Devesa S.S., Warren J.L., Fraumeni J.F. (1999). Rising incidence of renal cell cancer in the United States. JAMA.

[19] Tamburrini S., Comune R., Lassandro G., Pezzullo F., Liguori C., Fiorini V., Picchi S.G., Lugarà M., Del Biondo D., Masala S. (2023). MDCT Diagnosis and Staging of Xanthogranulomatous Pyelonephritis. Diagnostics.

[20] Figueiredo G., O’Shea A., Neville G.M., Lee S.I. (2022). Rare Mesenchymal Tumors of the Pelvis: Imaging and Pathologic Correlation. Radiographics.

[21] Scialpi M., Scaglione M., Angelelli G., Lupattelli L., Resta M.C., Resta M., Rotondo A. (2004). Emergencies in the retroperitoneum: Assessment of spread of disease by helical CT. Eur. J. Radiol..

[22] Trovato P., Simonetti I., Morrone A., Fusco R., Setola S.V., Giacobbe G., Brunese M.C., Pecchi A., Triggiani S., Pellegrino G. (2024). Scientific Status Quo of Small Renal Lesions: Diagnostic Assessment and Radiomics. J. Clin. Med..

[23] Xue L.Y., Lu Q., Huang B.J., Li C.X., Yan L.X., Wang W.P. (2016). Differentiation of subtypes of renal cell carcinoma with contrast-enhanced ultrasonography. Clin. Hemorheol. Microcirc..

[24] Mueller-Peltzer K., Negrao de Figueiredo G., Graf T., Rübenthaler J., Clevert D.A. (2019). Papillary renal cell carcinoma in contrast-enhanced ultrasound (CEUS)—A diagnostic performance study. Clin. Hemorheol. Microcirc..

[25] Couvidat C., Eiss D., Verkarre V., Merran S., Corréas J.M., Méjean A., Hélénon O. (2014). Renal papillary carcinoma: CT and MRI features. Diagn. Interv. Imaging.

[26] Chiarello M.A., Mali R.D., Kang S.K. (2018). Diagnostic Accuracy of MRI for Detection of Papillary Renal Cell Carcinoma: A Systematic Review and Meta-Analysis. AJR Am. J. Roentgenol..

[27] Dilauro M., Quon M., McInnes M.D., Vakili M., Chung A., Flood T.A., Schieda N. (2016). Comparison of Contrast-Enhanced Multiphase Renal Protocol CT Versus MRI for Diagnosis of Papillary Renal Cell Carcinoma. AJR Am. J. Roentgenol..

[28] Campbell N., Rosenkrantz A.B., Pedrosa I. (2014). MRI phenotype in renal cancer: Is it clinically relevant?. Top. Magn. Reason. Imaging.

[29] Castaldo R., Garbino N., Cavaliere C., Incoronato M., Basso L., Cuocolo R., Pace L., Salvatore M., Franzese M., Nicolai E. (2022). A Complex Radiomic Signature in Luminal Breast Cancer from a Weighted Statistical Framework: A Pilot Study. Diagnostics.

[30] Egbert N.D., Caoili E.M., Cohan R.H., Davenport M.S., Francis I.R., Kunju L.P., Ellis J.H. (2013). Differentiation of papillary renal cell carcinoma subtypes on CT and MRI. Am. J. Roentgenol..

[31] Kim J.K., Kim T.K., Ahn H.J., Kim C.S., Kim K.R., Cho K.S. (2002). Differentiation of subtypes of renal cell carcinoma on helical CT scans. Am. J. Roentgenol..

[32] Lee-Felker S.A., Felker E.R., Tan N., Margolis D.J., Young J.R., Sayre J., Raman S.S. (2014). Qualitative and quantitative MDCT features for differentiating clear cell renal cell carcinoma from other solid renal cortical masses. Am. J. Roentgenol..

[33] Pierorazio P.M., Hyams E.S., Tsai S., Feng Z., Trock B.J., Mullins J.K., Johnson P.T., Fishman E.K., Allaf M.E. (2013). Multiphasic enhancement patterns of small renal masses (≤4 cm) on preoperative computed tomography: Utility for distinguishing subtypes of renal cell carcinoma, angiomyolipoma, and oncocytoma. Urology.

[34] Ruppert-Kohlmayr A.J., Uggowitzer M., Meissnitzer T., Ruppert G. (2004). Differentiation of renal clear cell carcinoma and renal papillary carcinoma using quantitative CT enhancement parameters. Am. J. Roentgenol..

[35] Yang C.W., Shen S.H., Chang Y.H., Chung H.J., Wang J.H., Lin A.T., Chen K.K. (2013). Are there useful CT features to differentiate renal cell carcinoma from lipid-poor renal angiomyolipoma?. Am. J. Roentgenol..

[36] Zhang J., Lefkowitz R.A., Ishill N.M., Wang L., Moskowitz C.S., Russo P., Eisenberg H., Hricak H. (2007). Solid renal cortical tumors: Differentiation with CT. Radiology.

[37] Pooler B.D., Pickhardt P.J., O’Connor S.D., Bruce R.J., Patel S.R., Nakada S.Y. (2012). Renal cell carcinoma: Attenuation values on unenhanced CT. Am. J. Roentgenol..

[38] Yamamoto T., Gulanbar A., Hayashi K., Kohno A., Komai Y., Yonese J., Matsueda K., Inamura K. (2021). Is hypervascular papillary renal cell carcinoma present?. Abdom. Radiol..

[39] Marcon J., Graser A., Horst D., Casuscelli J., Spek A., Stief C.G., Reiser M.F., Rübenthaler J., Buchner A., Staehler M. (2020). Papillary vs clear cell renal cell carcinoma. Differentiation and grading by iodine concentration using DECT-correlation with microvascular density. Eur. Radiol..

[40] Fiordelisi M.F., Auletta L., Meomartino L., Basso L., Fatone G., Salvatore M., Mancini M., Greco A. (2019). Preclinical Molecular Imaging for Precision Medicine in Breast Cancer Mouse Models. Contrast Media Mol. Imaging.

[41] Khan O.S., Yunas M., Ijaz N., Iftikhar S., Toru H.K. (2022). Papillary Renal Cell Carcinoma as an Abdominal Cystic Mass. J. Ayub Med. Coll. Abbottabad.

[42] Banno T., Takagi T., Kondo T., Yoshida K., Iizuka J., Okumi M., Ishida H., Morita S., Nagashima Y., Tanabe K. (2020). Computed tomography imaging characteristics of clear cell papillary renal cell carcinoma. Int. Braz. J. Urol..

[43] Scaglione M., Masala S., Tamburrini S., Lassandro G., Barbuto L., Romano L., Iacobellis F., Sica G., Crivelli P., Turilli D. (2024). Abdominal Emergencies in Cancer Patients. Can. Assoc. Radiol. J..

[44] Rysz J., Franczyk B., Ławiński J., Gluba-Brzózka A. (2021). Characteristics of Clear Cell Papillary Renal Cell Carcinoma (ccpRCC). Int. J. Mol. Sci..

[45] Mendhiratta N., Muraki P., Sisk A.E., Shuch B. (2021). Papillary renal cell carcinoma: Review. Urol. Oncol..

[46] Ruebenthaler J., Reimann R., Hristova P., Staehler M., Reiser M., Clevert D.A. (2015). Parametric imaging of clear cell and papillary renal cell carcinoma using contrast-enhanced ultrasound (CEUS). Clin. Hemorheol. Microcirc..

